# Structural Neural Correlates of Double Decision Performance in Older Adults

**DOI:** 10.3389/fnagi.2020.00278

**Published:** 2020-09-02

**Authors:** Jessica N. Kraft, Andrew O’Shea, Alejandro Albizu, Nicole D. Evangelista, Hanna K. Hausman, Emanuel Boutzoukas, Nicole R. Nissim, Emily J. Van Etten, Pradyumna K. Bharadwaj, Hyun Song, Samantha G. Smith, Eric Porges, Steven DeKosky, Georg A. Hishaw, Samuel Wu, Michael Marsiske, Ronald Cohen, Gene E. Alexander, Adam J. Woods

**Affiliations:** ^1^Center for Cognitive Aging and Memory Clinical Translational Research, McKnight Brain Institute, University of Florida, Gainesville, FL, United States; ^2^Department of Neuroscience, College of Medicine, University of Florida, Gainesville, FL, United States; ^3^Department of Clinical and Health Psychology, College of Public Health and Health Professions, University of Florida, Gainesville, FL, United States; ^4^Department of Neurology, Perelman School of Medicine, University of Pennsylvania, Philadelphia, PA, United States; ^5^Brain Imaging, Behavior and Aging Laboratory, Department of Psychology and Evelyn F. McKnight Brain Institute, University of Arizona, Tucson, AZ, United States; ^6^Department of Neurology, College of Medicine, University of Florida, Gainesville, FL, United States; ^7^Department of Psychiatry, Neuroscience and Physiological Sciences Graduate Interdisciplinary Programs, and BIO5 Institute, University of Arizona and Arizona Alzheimer’s Consortium, Tucson, AZ, United States; ^8^Department of Biostatistics, College of Public Health and Health Professions, University of Florida, Gainesville, FL, United States

**Keywords:** speed of processing, UFOV, useful field of view, double decision, cognitive aging, structural MRI

## Abstract

Speed of processing is a cognitive domain that encompasses the speed at which an individual can perceive a given stimulus, interpret the information, and produce a correct response. Speed of processing has been shown to decline more rapidly than other cognitive domains in an aging population, suggesting that this domain is particularly vulnerable to cognitive aging (Chee et al., 2009). However, given the heterogeneity of neuropsychological measures used to assess the domains underpinning speed of processing, a diffuse pattern of brain regions has been implicated. The current study aims to investigate the structural neural correlates of speed of processing by assessing cortical volume and speed of processing scores on the POSIT Double Decision task within a healthy older adult population (*N* = 186; mean age = 71.70 ± 5.32 years). T1-weighted structural images were collected via a 3T Siemens scanner. The current study shows that less cortical thickness in right temporal, posterior frontal, parietal and occipital lobe structures were significantly associated with poorer Double Decision scores. Notably, these include the lateral orbitofrontal gyrus, precentral gyrus, superior, transverse, and inferior temporal gyrus, temporal pole, insula, parahippocampal gyrus, fusiform gyrus, lingual gyrus, superior and inferior parietal gyrus and lateral occipital gyrus. Such findings suggest that speed of processing performance is associated with a wide array of cortical regions that provide unique contributions to performance on the Double Decision task.

## Introduction

Older adults over the age of 65 are one of the largest and fastest growing populations in the United States (Goode et al., 1998). Numerous studies have shown that, even within healthy older adults, declines in cognition occur in numerous domains, including memory, executive functioning, and speed of processing (Goode et al., 1998). Speed of processing is the rate at which an individual can perceive a given stimulus (through visual perceptual components), interpret the information (through cognitive components), and produce a correct response (through psychomotor speed) (Salthouse, 2000). Importantly, speed of processing has been shown to decline more rapidly than other cognitive domains in the context of aging, suggesting that this domain may be particularly vulnerable to the deleterious effects of aging (Park et al., 2002; Finkel et al., 2007; Chee et al., 2009). In addition, speed of processing is a significant contributor to multiple domains of cognitive function (Salthouse, 1996). Prior research suggests that speed of processing may underlie age-related declines in memory, attention, reasoning and spatial abilities (Salthouse and Ferrer-Caja, 2003). Numerous studies to date suggest that age-related declines in speed of processing are significantly associated with a loss of functional instrumental activities of daily living (IADL) as demonstrated by poorer timed IADL scores, higher self-reported difficulties in IADL, poorer everyday problem solving, greater medical expenditures and greater motor vehicular crash risk (Goode et al., 1998; Edwards et al., 2005, 2014; Wolinsky et al., 2009; Bezdicek et al., 2016; Aust and Edwards, 2017). Given the substantial literature regarding the impact of speed of processing declines on functional abilities, there is a profound need for effective interventions for this cognitive domain.

### Interventions for Speed of Processing

Attempts have been made to ameliorate declines through the use of standardized cognitive training. Several randomized controlled trials have shown the efficacy of domain-specific cognitive training on improving speed of processing scores with moderate to large effect sizes (Ball et al., 2002; Vance et al., 2007; Simpson et al., 2012; Wolinsky et al., 2013). Notably, this is not the case with other domains, such as executive functioning, attention, and language, which show little to no improvements within domain-specific cognitive training (Lampit et al., 2014). Collectively, this suggests that speed of processing is a cognitive domain susceptible to decline in aging, but such declines may be reversed through cognitive training paradigms.

Some of the most successful randomized controlled trials aimed at improving speed of processing have utilized Useful Field of View (UFOV) cognitive training (Ball et al., 2002). In this paradigm, the participant is asked to identify a central target with subsequent subtasks including the addition of a peripheral target and increasing number of distractors in the periphery. Difficulty is adjusted by altering presentation time of the stimuli and through the addition of distractors, thus targeting cognitive components related to speed of processing. The Advanced Cognitive Training for Independent and Vital Elderly (ACTIVE) study, a large (*n* = 2802) randomized controlled trial using the UFOV task in a 6-week intervention, found improvements in speed of processing lasting up to ten years (Ball et al., 2002; Edwards et al., 2017). Notably, UFOV-based interventions demonstrated ‘near transfer’ effects to other speed of processing assessments (Wolinsky et al., 2013). However, studies have failed to find ‘far transfer’ effects to other cognitive domains such as attention (Wolinsky et al., 2013), memory (Edwards et al., 2002, 2005) and executive function (Edwards et al., 2005, 2018).

Age-related cognitive decline has been associated with numerous deleterious functional outcomes, including loss of physical function, poorer subjective quality of life, and greater difficulties in activities of daily living in older adults (Anton et al., 2015; Ryu et al., 2016). In-light of these findings, several studies have shown that UFOV training interventions are efficacious in improving real-world outcomes following UFOV training interventions. Specifically, UFOV training interventions have shown improvements in timed IADL tasks and self-reported improvements in IADLs (Edwards et al., 2002, 2005). Longitudinal follow-up of the large ACTIVE cohort demonstrated that each UFOV training session was associated with a 10% reduction in dementia prevalence even 10 years following the intervention (Edwards et al., 2017). Additionally, recent research has shown that performance on UFOV-based interventions is a significant predictor for motor vehicle accidents in an older adult population, a key factor in functional independence in older adults (Ball et al., 2006; Edwards et al., 2006, 2009). Given the long-lasting intervention effects, including improvements in speed of processing, functional outcomes, and reductions in dementia prevalence, the UFOV task remains an essential target of cognitive interventions.

Useful Field of View training was recently adapted and made commercially available by POSIT Science Brain HQ^1^ and retitled the Double Decision task. This task is administered through an online portal with updated visual aesthetics and inclusion of a within-task adaptive change in difficulty based on participant performance. Like UFOV, Double Decision asks participants to identify a target in the center of the screen while also attending to a target in the periphery among distractor stimuli. Adaptive change in difficulty is implemented by manipulating both the visual perceptual components of speed of processing (by adjusting the speed of stimulus display) and the cognitive components of speed of processing (by adjusting the similarity of the targets and distractors). At present, the Double Decision training paradigm serves as the prevalent implementation for UFOV training in recent and ongoing clinical trials. This paradigm has been used in a number of recent speed of processing interventions and has been efficacious in improving speed of processing performance in older adults (Edwards et al., 2013; Kaur et al., 2014; Lin et al., 2016; Ross et al., 2018).

### Neural Correlates of UFOV/Double Decision Performance and Speed of Processing

Studies looking at a broad range of speed of processing paradigms have found significant associations with a variety of markers of neuronal integrity. Specifically, decreased cortical volume has been associated with poorer speed of processing performance within the bilateral precentral gyrus, bilateral inferior frontal gyrus, left superior frontal gyrus, bilateral superior parietal regions, and bilateral middle frontal gyrus (Chee et al., 2009; Hong et al., 2015). Additionally, greater hippocampal volume has been associated with better speed of processing, suggesting hippocampal volume may be especially related to speed of processing performance (Papp et al., 2014; O’Shea et al., 2016; Tsapanou et al., 2019). Notably, O’Shea et al. (2016) found that hippocampal volume accounts for 11% of the variance in speed of processing scores.

Although speed of processing training using UFOV/Double Decision is one of the more effective forms of cognitive training to date, very few studies have investigated the neural correlates of the UFOV/Double Decision task itself. Two functional magnetic resonance imaging (fMRI) studies utilizing the UFOV task in-scanner have shown significant blood-oxygen level dependent (BOLD) activation of numerous areas, including the anterior cingulate cortex, supplementary motor area, dorsolateral prefrontal cortex (DLPFC), insula, precuneus, temporoparietal junction, and visual cortices (Scalf et al., 2007; Ross et al., 2018).

Only one small study to date (*n* = 41) has investigated the role of cortical thickness in UFOV performance (Schmidt et al., 2016). This elegantly designed study found that greater cortical thickness in the bilateral intraparietal sulcus, frontal cortex, precuneus, midcingulate, inferior parietal lobule, and dorsolateral prefrontal cortex were all associated with greater UFOV performance (Schmidt et al., 2016). These aforementioned regions of interest comprise the fronto-parietal control network, an adaptive control functional connectivity neural network (Dosenbach et al., 2007; Schmidt et al., 2016). Understanding the neural underpinnings of the UFOV cognitive training intervention could provide unique insight into potential avenues for intervention optimization (e.g., adjunctive non-invasive brain stimulation targeting involved regions). The present study sought to further determine the gray matter structural neural correlates of Double Decision task performance in older adults, by using a vertex-wise analysis approach. To achieve this goal, 186 participants performed the Double Decision task prior to undergoing T1-weighted magnetic resonance imaging. Based on prior findings regarding the structural (Schmidt et al., 2016) and functional (Ross et al., 2018) neural underpinnings of UFOV performance, we hypothesized that poorer Double Decision performance is related to cortical degradation in the dorsolateral prefrontal cortex, insula, inferior parietal lobule, supplementary motor area, anterior cingulate cortex, and precuneus.

## Materials and Methods

### Participants

A sample of healthy older adults (*N* = 186) were recruited at the University of Florida, Gainesville FL, and University of Arizona, Tucson, AZ, United States as part of two ongoing randomized clinical trials with identical inclusion/exclusion criteria (R01AG054077; K01AG050707). Participants were between the ages of 65–88 years [113 females, mean education = 16.13 years (SD ± 2.39), mean age = 71.70 years (SD ± 5.32)]. The full details of inclusion/exclusion criteria are described in a previous publication (Woods et al., 2018). Briefly, participants were excluded if they: (a) were outside the age range of 65–89, (b) had a history of neurological or psychiatric disorders, (c) had MRI contraindications, (d) were left-handed, or (e) had any current substance abuse or dependence problem. General cognitive ability was assessed using the Uniform Data Set (UDS 3.0) of the National Alzheimer’s Coordinating Center (NACC) (Weintraub et al., 2009). The UDS serves as a comprehensive neuropsychological battery for screening individuals for possible dementia and mild cognitive impairment (MCI), and is normed based on age, education, and sex (Shirk et al., 2011). Possible MCI was defined as 1.5 standard deviations (SD) below the mean for age, sex, and education normed scores in any of the following five domains: general cognition, memory, visuospatial, executive functioning/working memory, or language. Participants meeting criteria for possible MCI were subsequently excluded from the study. Participants were excluded following an in-person screening visit if they were color-blind, had impaired vision (defined as uncorrected vision worse than 20/80) or notable hearing loss (defined as an inability to hear a target at 20dB or louder with background noise). Additionally, any participants with abnormal findings in their MRI brain scans were excluded from the study (i.e., cyst, tumors, etc.). The University of Florida and University of Arizona Institutional Review Boards approved all study protocols. Participants provided written informed consent prior to any study procedures.

### Brain HQ- Double Decision Task

**FIGURE 1:**
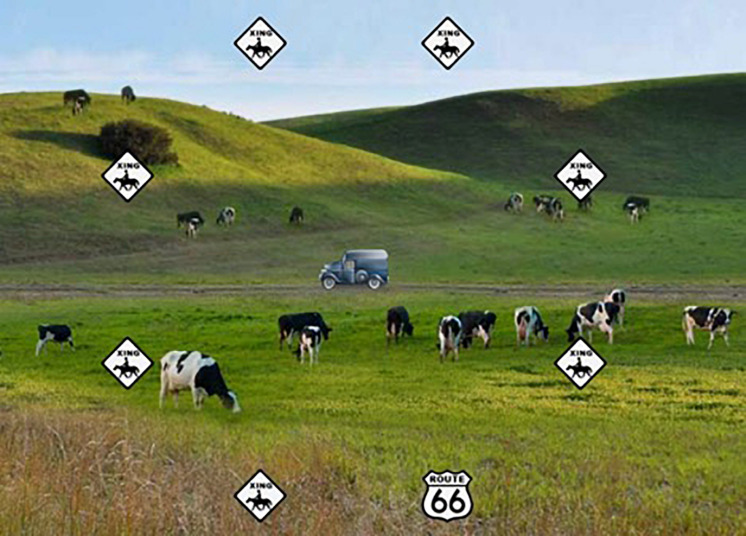
Example of the POSIT Double Decision paradigm. Reproduced/adapted from POSIT Brain HQ, used with permission.

The POSIT Brain HQ Double Decision task has been widely used in adaptive cognitive training paradigms and was therefore selected as a measure of speed of processing (Edwards et al., 2013; Lin et al., 2016; Ross et al., 2018). The task requires participants to identify which central target was presented on the screen (either a car or a truck) and the location of the peripheral target (a Route 66 sign) (Figure 1). Possible presentation times in milliseconds (ms) ranged from 32 ms to 2600 ms and there were 25 presentations total. The number of possible distractors ranged from 0 to 47. Performance was set at a moderate level of difficulty with a starting presentation time of 501ms and 7 distractors. As this is an adaptive training paradigm, the targets were presented for variable lengths of time depending on participant accuracy, with longer presentation times following incorrect trials and shorter presentation times following correct trials. Participants’ scores were calculated as the average presentation time of correct responses in log milliseconds, as reaction time is positively skewed. Lower scores on the task equated to faster speed of processing scores.

### MRI Acquisition

T1-weighted magnetization prepared rapid gradient echo (MPRAGE) structural scans were collected via a Siemens 3T Prisma scanner using a 64-channel head coil (University of Florida site, *n* = 129) or via a Siemens 3T Skyra scanner using a 32-channel head coil (University of Arizona site, *n* = 57) (Siemens, Erlangen, Germany). T1- weighted MPRAGE scans were collected with the following parameters at both sites: repetition time (TR) = 1800 ms; echo time (TE) = 2.26ms; flip angle = 8°; field of view = 256mm × 256mm × 176mm; voxel size = 1mm^3^.

### Structural Neuroimaging Processing

Cortical surface area and cortical thickness from T1-weighted images were analyzed via the FreeSurfer pipeline (version 6.0), the details of which are described elsewhere (Dale et al., 1999; Fischl et al., 1999, 2002; Fischl and Dale, 2000). This FreeSurfer processing pipeline involves removal of non-brain tissue, volumetric labeling of white matter and subcortical gray matter structures, surface tessellation, intensity normalization of the T1-weighted image, segmentation of white matter, surface atlas registration, surface extraction, and gyral labeling (Desikan et al., 2006). Following FreeSurfer processing, output was visually assessed and manual corrections for any segmentation errors were performed as needed. Specific manual edits included addition of control points to extend the white matter surface and reconstruction edits to remove non-cortical matter that was erroneously included in the gray matter surface. The corrections were then re-ran through the same FreeSurfer recon-all pipeline prior to data analyses. Cortical thickness was defined within the FreeSurfer pipeline at each vertex as the distance (in mm) from the gray/white matter boundary to the pial surface, whereas cortical surface area was designated as an area (in mm^2^) equal to the average of surrounding triangles around each individual vertex on the gray/white boundary surface tessellation.

### Neuroimaging Statistical Analyses

This study examined both cortical surface area and cortical thickness via a vertex-wise analysis using the QDEC FreeSurfer tool. Vertex-wise cortical surface area and thickness values were mapped onto the normalized, tessellated cortical surface, which was smoothed with a 20-mm full width at half maximum kernel (Lampit et al., 2015; Suárez-González et al., 2016). QDEC creates general linear models (GLM) to assess cortical morphological differences between subjects using a mass univariate approach. Education, sex, and scanner location were included in our original model as covariates, with Double Decision scores as our independent variable and cortical thickness and surface area as the dependent variables.

As we are primarily focused on the effects of speed of processing performance in the context of aging, we chose not to include age as a covariate. By including age as a covariate in our original model, this would assess speed of processing performance irrespective of age, or holding age constant. However, to assess the impact of age on the association between Double Decision scores and cortical thickness and surface area, we conducted a secondary analysis adding age as a covariate in our original model. Additionally, we assessed an age by Double Decision score interaction term to assess how age may moderate the relationship between Double Decision scores and cortical thickness.

To account for multiple comparisons associated with a mass univariate approach, we set a false discovery rate (FDR) threshold at *p* < 0.05 (Benjamini and Hochberg, 1995). Percent overlap in a specific region of interest (ROI) was calculated by comparing the number and location of individual significant vertices indicated in QDEC, to MNI coordinates. ROIs were defined via the Desikan-Killiany atlas, a well-validated, well-defined cortical atlas (Desikan et al., 2006). Percent overlap was defined as the number of significant vertices within an ROI divided by the total number of vertices in that ROI. This method allows us to quantify and pinpoint decreased cortical thickness associated with poorer Double Decision performance within otherwise exceedingly large ROIs. In addition, we performed an additional validation analysis to compare and contrast results using an alternative multiple comparison approach: Monte Carlo simulation. This serves as an additional validation of the consistency of findings from the current study. We also performed a cross-validation analyses using a split-half reliability analysis where participants and randomly assigned to two groups and analyses re-run to evaluate reliability and consistency of findings within the sample. These analyses were performed at *p* < 0.01, as the sample size/power were effectively halved to perform the cross-validation analysis.

## Results

**FIGURE 2:**
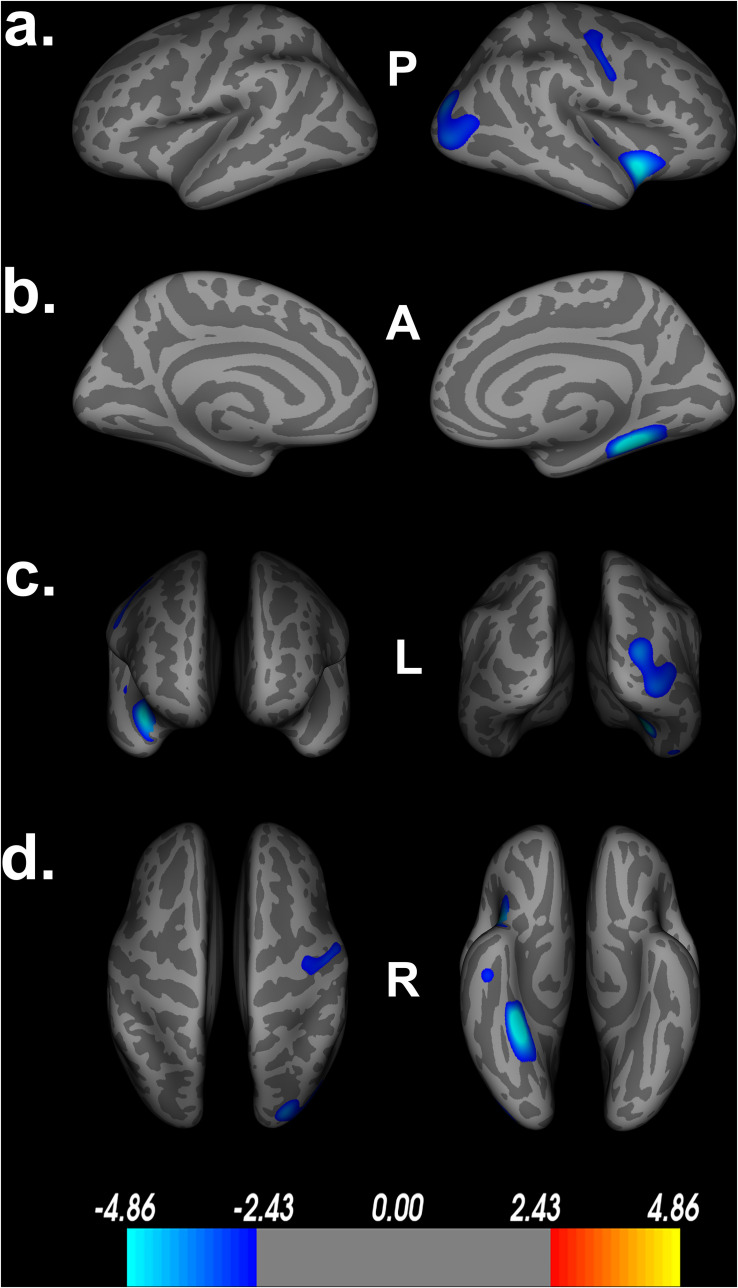
Decreased cortical thickness related to POSIT Double Decision scores. **(a)** left and right hemisphere lateral views, respectively. **(b)** left and right hemisphere medial views, respectively. **(c)** anterior and posterior views, respectively. **(d)** superior and inferior views, respectively. Model covariates: sex, education, scanner. P- posterior, A- anterior, L- left, R- right.

**FIGURE 3:**
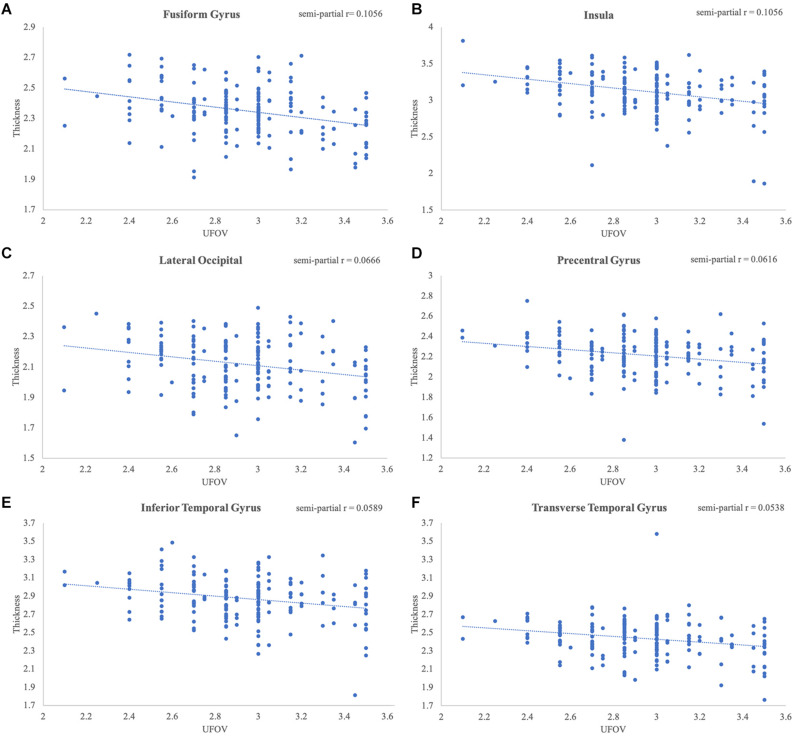
Significant relationships between Double Decision scores and cortical thickness. Scatterplots represent the relationship at the most significant vertex within the **(A)** fusiform gyrus, **(B)** insula, **(C)** lateral occipital gyrus, **(D)** precentral gyrus, **(E)** inferior temporal gyrus and **(F)** transverse temporal gyrus, controlling for education, sex and scanner covariates.

Poorer Double Decision scores were significantly associated with less cortical thickness in several brain regions within the right posterior frontal, temporal, parietal, and occipital lobes (FDR < 0.05) (Figure 2). Cluster size, p-values (uncorrected), and peak vertex coordinates in MNI space for all significant regions that survived FDR corrections can be found in Table 1. Notably, significant regions of interest (ROI) clusters span and encompass large portions of numerous Desikan-Killiany atlas-based regions. Scatter plots of Double Decision scores and cortical thickness at the MNI coordinates listed in Table 1, can be found in Figure 3. These scatter plots, and corresponding semi partial *r*-squared statistics, controlling for covariates (sex, education, scanner) represent the relationship between cortical thickness and Double Decision score at the most significant vertex within the ROI clusters.

**TABLE 1:** Significant cortical thickness ROIs.

Brain Region (*ROIs encompassed)*	Size (mm^2^)	Percent of ROI encompassed	X	Y	Z	P-uncorrected
**Right Hemisphere Thickness**						
Fusiform gyrus	1104.94		37.2	−41.9	−13.0	< 0.0001
*Fusiform gyrus*		*21.50%*				
*Lingual gyrus*		*11.12%*				
*Parahippocampal gyrus*		*37.66%*				
Insula	786.81		38.1	−4.0	−12.3	< 0.0001
*Insula*		*25.76%*				
*Superior temporal gyrus*		*6.99%*				
*Lateral orbitofrontal gyrus*		*2.82%*				
*Temporal pole*		*6.12%*				
Lateral occipital gyrus	1576.05		25.8	−85.2	15.0	0.0001
*Lateral occipital gyrus*		*31.96%*				
*Inferior parietal lobule*		*3.46%*				
*Superior parietal lobule*		*0.60%*				
Precentral gyrus	544.47		48.9	−5.5	28.2	0.0007
*Precentral gyrus*		*13.00%*				
Inferior temporal gyrus	116.87		46.1	−14.3	−34.3	0.0009
*Inferior temporal gyrus*		*4.48%*				
Transverse temporal gyrus	24.88		49.7	−14.6	1.3	0.0022
*Transverse temporal gyrus*		*5.63%*				
*Superior temporal gyrus*		*0.13%*				

*Underlined ROIs are significant decreased cortical thickness and are labeled based on the location of the most significant vertex. Italicized ROIs are the Desikan-Killiany ROIs encompassed within the significant decreased cortical thickness clusters. All listed values survived FDR corrections.*

Additionally, Table 1 describes smaller Desikan-Killiany ROIs encompassed by the large significant clusters. We observed lateralized right hemisphere decreased cortical thickness in the superior, transverse, and inferior temporal gyrus, insula, fusiform gyrus, lateral orbitofrontal gyrus, precentral gyrus, parahippocampal gyrus, lingual gyrus, temporal pole, superior and inferior parietal gyrus and lateral occipital gyrus. In the left hemisphere, there were no significant relationships between cortical thickness and Double Decision scores that survived FDR correction. Within both hemispheres, there was not a significant relationship between cortical surface area and Double Decision scores that survived FDR correction.

### Impact of Covariates on the Model

**FIGURE 4:**
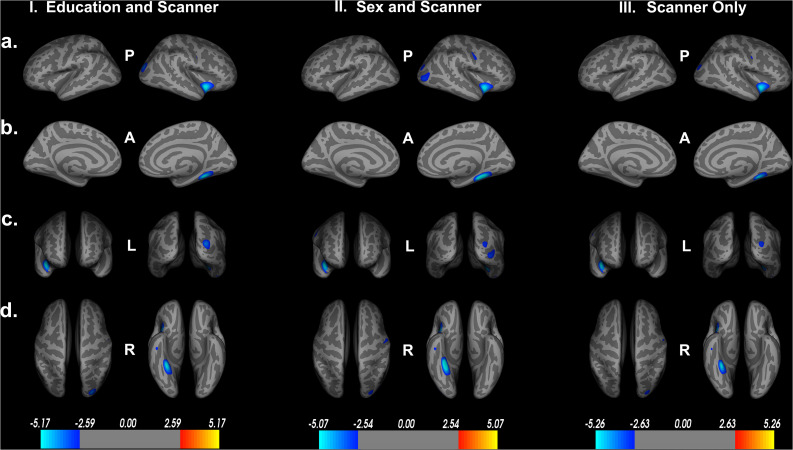
Decreased cortical thickness related to POSIT Double Decision scores for: I. education and scanner covariates only, II. sex and scanner covariates only and III. scanner covariate only. (row **a**) left and right hemisphere lateral views, respectively. (row **b**) left and right hemisphere medial views, respectively. (row **c**) anterior and posterior views, respectively. (row **d**) superior and inferior views, respectively. P- posterior, A- anterior, L- left, R- right.

To assess the impact of covariates on cortical thickness findings, we reanalyzed the relationship between Double Decision scores and cortical thickness with our initial covariates excluded. Figure 4 demonstrates the relationship between Double Decision scores and cortical thickness hierarchically including covariates into the model. We see a decrease in the size and number of significant ROIs in all permutations compared to the original model. Additionally, we see a larger range in each of the scales compared to the original model needed to achieve statistical significance following FDR corrections. This suggests that the covariates in the final model are accounting for a significant portion of the variance associated with Double Decision scores, compared to the models with the covariates excluded.

### Impact of Age on the Model

**FIGURE 5:**
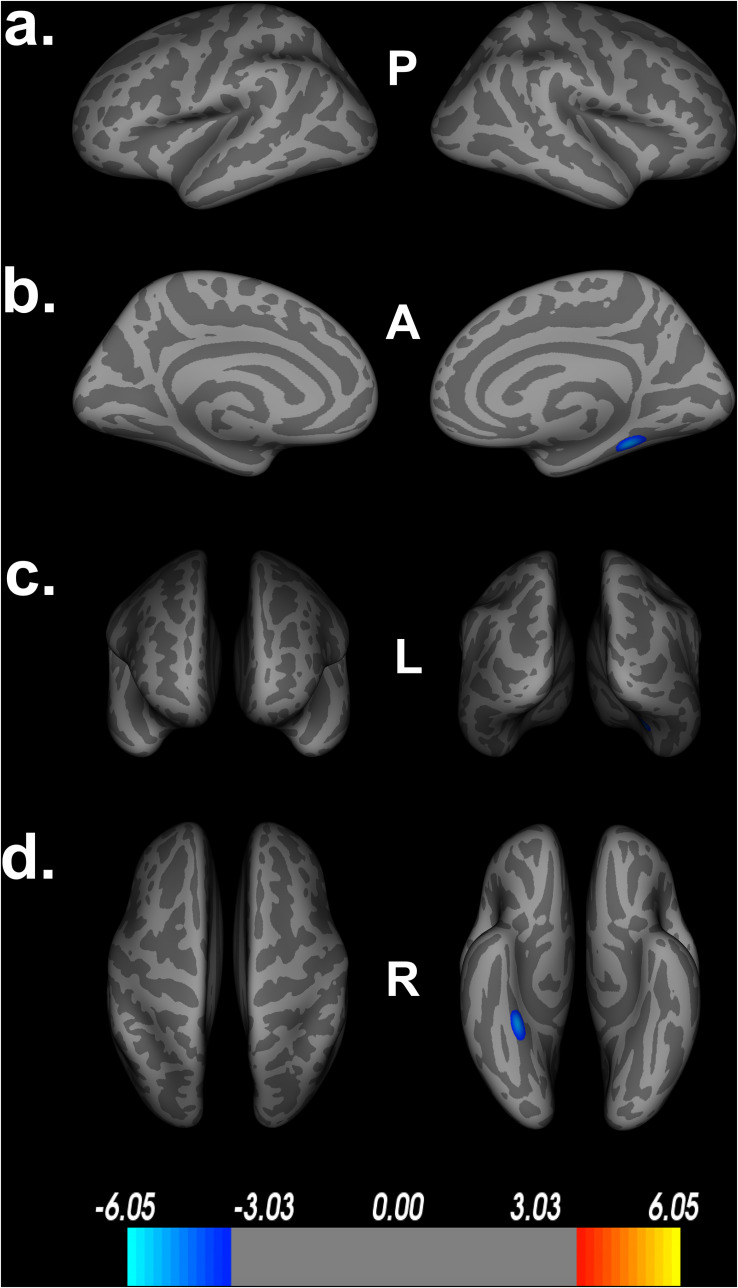
Decreased cortical thickness related to POSIT Double Decision scores, covarying age, sex, education and scanner. **(a)** left and right hemisphere lateral views, respectively. **(b)** left and right hemisphere medial views, respectively. **(c)** anterior and posterior views, respectively. **(d)** superior and inferior views, respectively. P- posterior, A- anterior, L- left, R- right.

Within our secondary analyses, covarying out age, sex, education and scanner, we see that only one large cluster, spanning the right fusiform gyrus, parahippocampal gyrus and lingual gyrus survive FDR correction (Figure 5). These regions are among the largest ROIs implicated in our original model, suggesting that these regions may serve as core features of Double Decision performance outside the context of aging. Similar to the covariates above, we see a decrease in the size and number of significant ROIs and a larger range in the scale needed to achieve statistical significance following FDR corrections. Again, this suggests that including age into the model adds explains additional significant variance in the relationship between Double Decision scores and cortical thickness. Additionally, we found no significant age by Double Decision score interaction.

### Validation of FDR vs. Monte Carlo Simulations as Methods for Multiple Comparison Correction

**FIGURE 6:**
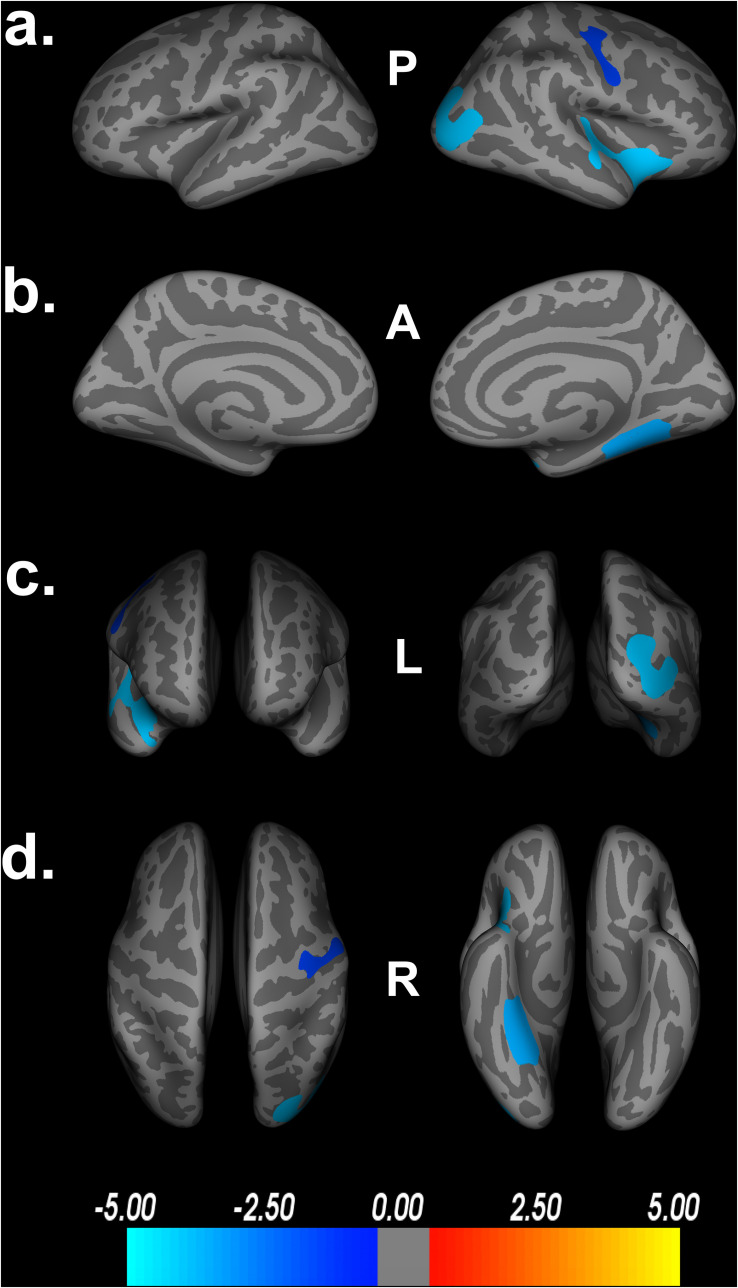
Decreased cortical thickness related to POSIT Double Decision scores using Monte Carlo simulations, set at 10,000 iterations and a cluster-wise threshold *p* = 0.05. **(a)** left and right hemisphere lateral views, respectively. **(b)** left and right hemisphere medial views, respectively. **(c)** anterior and posterior views, respectively. **(d)** superior and inferior views, respectively. P- posterior, A- anterior, L- left, R- right.

To further validate the FDR corrected findings, we used Monte Carlo simulations, set at 10,000 iterations and a cluster-wise *p*-threshold = 0.05. Comparison of the Monte Carlo simulations (Figure 6) to the traditional FDR findings in Figure 1 shows little difference between the two multiple comparison corrections. Specifically, we see that the Monte Carlo simulations found decreased cortical thickness in the lingual gyrus, which was not observed in the original model with FDR corrections. Additionally, we find slightly larger areas of decreased cortical thickness in the fusiform gyrus and superior temporal gyrus in relation to Double Decision scored, compared to the FDR-corrected model. No regions became insignificant following Monte Carlo corrections. Thus, findings using Monte Carlo appear to be slightly more liberal than FDR correction, but were consistent overall.

### Replicability and Reliability of Results With the Sample

To test the internal consistency and reliability of our findings, we performed a split-half reliability analysis. This consists of randomly dividing our sample of participants into two equal groups and rerunning the same QDEC vertex-wise analyses on both groups. Assessing demographic information, there was no significant differences between the groups on age [*t*(184) = 1.576, *p* = 0.117], years of education [*t*(184) = −0.615, *p* = 0.539] or UFOV performance [*t*(184) = −0.750, *p* = 0.454]. There was no significant difference in the proportion of male to female between the two groups χ^2^(*df* = 1, *n* = 186) = 0.203, *p* = 0.764. Similarly, there was no significant difference in the proportion of participants located at each study site between the two groups χ^2^(*df* = 1, *n* = 186) = 0.228, *p* = 0.751. Table 2 shows the breakdown of the demographic information by group.

**TABLE 2:** Group specific demographics.

	Group 1 (*n* = 93)	Group 2 (*n* = 93)	Combined (*n* = 186)
Age mean (SD),	72.28 (5.27)	71.05 (5.34)	71.70 (5.32)
Education mean (SD)	16.02 (2.48)	16.24 (2.29)	16.13 (2.39)
Sex, female	55	58	113
Scanner location, UF	66	63	129
UFOV performance	2.92 (0.32)	2.96 (0.29)	2.94 (0.30)

**FIGURE 7:**
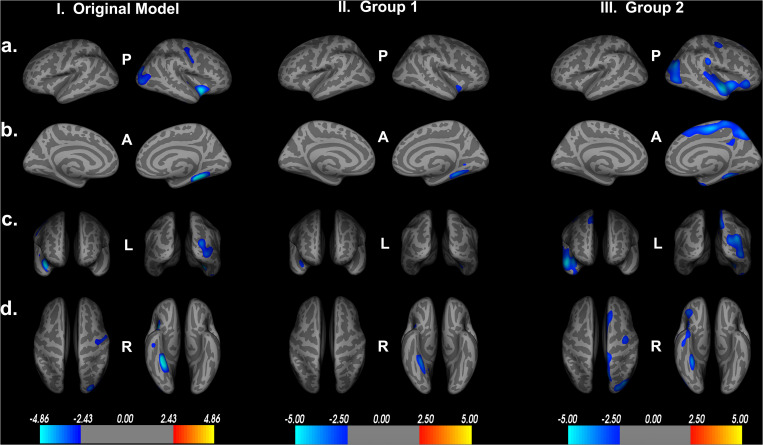
Random group assignment cortical thickness findings (*n* = 93 for each group; total *n* = 186). Significant clusters set at *p* > 0.01. **(a)** left and right hemisphere lateral views, respectively. **(b)** left and right hemisphere medial views, respectively. **(c)** anterior and posterior views, respectively. **(d)** superior and inferior views, respectively. P- posterior, A- anterior, L- left, R- right.

We then reran the QDEC analyses with identical input parameters to assess right lateralized cortical thickness differences between the two groups. Education, sex, and scanner location were again included as covariates within each group, and threshold for significant findings was set at *p* < 0.01. We see the cortical thickness pattern of findings from the overall model is similar to both groups 1 and 2 (Figure 7). Based on the random grouping assignment, we see decreased whole brain cortical thickness in group 2 compared to group 1. However, in both groups, we see that less cortical thickness in the fusiform gyrus, parahippocampal gyrus, lingual gyrus, insula and superior temporal gyrus are significantly related to poorer Double Decision performance, controlling for covariates. These regions are not only identical to the original model, but are also the regions with the highest percent of ROI encompassed in the original model (Table 1). This suggests that, although group 2 has less cortical thickness overall, decreased cortical thickness in the fusiform gyrus, parahippocampal gyrus, lingual gyrus, insula and superior temporal gyrus within each group are core regions associated with poorer Double Decision performance. This random group assignment cross validation analysis demonstrates that our findings in the overall model were replicable and reliable within the sample.

## Discussion

This study aimed to investigate the structural neural correlates of the Double Decision task. We found extensive cortical thinning associated with poorer performance on the Double Decision task throughout the right hemisphere. Notably, such findings are consistent with previous research investigating cortical thickness and UFOV performance in an ROI based analysis (Schmidt et al., 2016), and consistent with a broader speed of processing paradigms (Chee et al., 2009; MacPherson et al., 2017). Speed of processing is a complex and, perhaps, foundational ability that contributes to a variety of cognitive domains that decline with advanced age. Speed of processing performance commonly requires integration of sensory, motor, response inhibition, and attentional processing for execution, among other domains (Salthouse, 2000). For example, Double Decision task performance encompasses numerous aspects of cognition such as attention, brief visual memory, encoding and recognition of visual targets, rule maintenance, pattern comparison, and error recognition. As such, it is perhaps not surprising that Double Decision performance is associated with a broad array of cortical regions in the current study. These unique findings elucidate the structural neural correlates of Double Decision performance within a voxel-wise analysis. Hereafter, we describe the potential roles and implications of major structural findings from the current study.

### Right Lateralization of Findings

While prior functional studies of the UFOV/Double Decision paradigm found bilateral activation associated with performance, our findings demonstrate right lateralization of structures associated with performance (Ross et al., 2018). This pattern of findings is consistent with other cognitive domains within the context of aging. For example, prior work on age-related working memory decline demonstrates a pattern of right lateralized frontal structural contributions to performance (Nissim et al., 2019), whereas working memory performance in younger adults typically demonstrates bilateral patterns of activation in functional paradigms (e.g., Cabeza, 2002). Similar patterns have also been demonstrated in sustained attention (Mitko et al., 2019). These collective data may suggest that right hemisphere brain structures in the context of their role in these cognitive domains are particularly vulnerable to change with age. However, longitudinal studies of right hemisphere atrophy and change in performance on Double Decision, working memory and other domains (e.g., sustained attention) evidencing differential patterns of structural vs. functional involvement are needed to elucidate the validity of this hypothesis. Regardless, our data suggest that right lateralized structures are associated with differences in Double Decision performance in older adults. The subsequent discussion addresses our predominately right hemisphere findings.

### Right Frontal Lobe

Decreased cortical thickness in the lateral orbitofrontal gyrus was associated with poorer Double Decision scores. Studies have suggested that the lateral orbitofrontal gyrus, also known as the ventromedial prefrontal cortex is particularly important for error processing within cognitive tasks, and perturbations in this area are related to reduced inhibitory control (Krämer et al., 2013). Recent fMRI paradigms have shown activation in the lateral orbitofrontal cortex during tasks of spatial recognition and presentation of novel visual stimuli, suggesting that this area is especially sensitive to visual recognition and learning tasks (Rolls et al., 2005; Nee and D’Esposito, 2016). Numerous studies have also found that substantial connections exist between the lateral orbitofrontal gyrus and the inferior temporal gyrus, which are commonly implicated in visual and object recognition (Rolls et al., 2005; Rolls and Grabenhorst, 2008). Visual and object recognition is critical in Double Decision performance, as successful performance is mediated by recognition of both the central target and the location of the peripheral target. As such, the role of the orbitofrontal cortex is likely essential in Double Decision performance.

The precentral gyrus is responsible for motor execution and coordination and contains the supplementary motor area (SMA). Numerous fMRI paradigms have shown that this area is activated during motor tasks, and this region is engaged in anticipation of completing motor tasks (Sharma and Baron, 2014; Bazán et al., 2015). Studies have shown that not only was the precentral gyrus activated during a motor task and motor imagery tasks, significant BOLD activation was demonstrated in preparation for a motor tasks and in reaction time maintenance of a task (Berman et al., 2012). Additionally, within the most anterior portions of the precentral gyri lie the frontal eye fields, an area essential for visual attention and target tracking (Yeo et al., 2011). This region is highly interconnected with the inferior parietal lobule (IPL), a region that was also implicated in the current study (Dosenbach et al., 2007). Notably, decreased cortical thickness findings in the present study in relation to Double Decision scores encompassed 13% of the total precentral gyrus. Given the rapid presentation of stimuli within the Double Decision task, reduced precentral cortical thickness may suggest a slowing of response and decreased visual attention associated with poorer Double Decision performance.

### Right Temporal Lobe

Significant portions of the right temporal lobe were also associated with Double Decision performance. Specifically, cortical thinning of the right superior, inferior, and transverse temporal gyri was significantly associated with poorer Double Decision performance. The superior temporal gyrus is a large region containing the transverse temporal gyrus (also known as the primary auditory cortex) and Wernicke’s area (BA 22), the latter of which is responsible for sound perception and language comprehension (Woods et al., 2009). Significant findings in this region may be explained by the presentation of unique sounds for correct and incorrect trials within the Double Decision task. Additionally, recent fMRI research has found that the superior temporal gyrus is also activated during novel problem solving and insight, which are important for Double Decision performance (Tian et al., 2017; Lin et al., 2018). Indeed, prior research has suggested that decreased cortical thickness in the superior temporal gyrus is related to poorer performance in a number of cognitive domains, including attention and speed of processing (Achiron et al., 2012).

Cortical thinning was also observed in the right transverse temporal gyrus in relation to poorer Double Decision scores. This small region contains the primary auditory cortex (BA 41 and 42) and is responsible for auditory information processing. fMRI data has demonstrated that this area is activated during tasks of error recognition, specifically when participants are presented with unique sounds during correct and incorrect responses, which occurs in the Double Decision task (van Atteveldt et al., 2009; Weis et al., 2013; Linke and Cusack, 2015). This feature may account for cortical thinning associations specifically within the transverse temporal gyrus to Double Decision performance.

Cortical thinning within the inferior temporal gyrus was associated with poorer Double Decision performance in the present study. This region is within the ventral stream of visual processing, allowing for recognition of objects in our field of view, pattern recognition and spatial awareness (Ishai et al., 1999; Herath et al., 2001). These visual processes appear to contribute to better performance on the Double Decision task. Performance on the Double Decision task is dependent on differentiating the Route 66 sign from distractors and differentiating the central targets (a truck or a car) through slight differences in pattern and shape. Performance is also mediated by quick visual attention to areas in the periphery. Studies using intracerebral recordings demonstrated that neurons within inferior temporal gyrus were selectively activated during visual tasks and during recall of stimuli (Hamamé et al., 2012). The decreased cortical thickness findings in the present study establish the contribution of the inferior temporal gyrus in Double Decision tasks.

The right insula, including 25.76% of this region, demonstrated cortical thinning in relation to poorer Double Decision task performance. The insula is involved with numerous cognitive functions. Specifically, fMRI studies have shown that insula activation is involved in error processing (Menon et al., 2001; Cracco et al., 2016). The Double Decision task provides feedback on each trial for accuracy of both the central target and location of the peripheral target. Such feedback directs participants’ attention and may enhance participants’ strategies for successfully completing the task. Indeed, several studies have shown that the anterior insula is a hub for the cingulo-opercular network (CON), also known as the ventral attention network, which has been implicated in target detection, rule maintenance and error processing (Corbetta et al., 2000; Fox et al., 2006). In fact, the area of right insula implicated in the current study demonstrates 67.21% overlap with the defined anterior insula hub of the CON network, further supporting a potential involvement of CON in the Double Decision task (Yeo et al., 2011). The relationship between CON and Double Decision performance deserves future research to elucidate their relationship.

Significant cortical thinning in the right fusiform gyrus was associated with poorer Double Decision performance, representing 21.50% of the fusiform gyrus. Studies have shown that the fusiform gyrus plays a large role in processing color and resolution information within visual integration tasks (Dosenbach et al., 2007; Wang et al., 2013; Sokolov et al., 2018). Multimodal imaging research by Dosenbach et al. (2007) has demonstrated that the fusiform gyrus, along with a number of different regions, including the inferior parietal lobule, precuneus, frontal cortex were highly functionally connected, comprising the fronto-parietal control network (FPCN). Indeed, a considerable number of regions commonly associated with the FPCN network also demonstrated significant decreased cortical thickness associated with poorer Double Decision performance in the current study.

Our findings also include significant right hemisphere cortical thinning in the parahippocampal gyrus in relation to Double Decision scores. Notably, decreased cortical thickness encompassed 37.66% of the entire parahippocampal gyrus, more than any other ROI. The parahippocampal gyrus has been widely shown to be involved with memory encoding and retrieval (Takahashi et al., 2002; Xu et al., 2019). The parahippocampal place area (PPA) region of the parahippocampal gyrus is responsible for memory and encoding of visual features during presentation of a spatial task, which are heavily utilized in the Double Decision tasks. Emerging literature suggests that impairments within the parahippocampal place area are associated with markedly poorer spatial recognition and performance (Dundon et al., 2018; Murias et al., 2019).

The temporal pole is the most anterior cortical area in the temporal lobe and although less is known about this area, research has shown that this area communicates extensively with the amygdala (Li et al., 2016). Studies to date have shown that the temporal pole is implicated in encoding and retrieving unique auditory and visual stimuli (Waldron et al., 2015). Our significant findings within the right temporal pole is most likely associated with its role in brief memory retrieval of unique auditory and visual stimuli.

### Right Parietal Lobe

The superior parietal lobule is a cortical region that is strongly implicated in spatial orientation, visual search efficiency, and mental rotation tasks (Gogos et al., 2010; Bueichekú et al., 2015). Specifically, the right superior lobule has been shown to be involved in visual contextual processing and topographical representation of visual scenes (Lester and Dassonville, 2014). Successful performance on the Double Decision task depends on rapid processing of both a central and peripheral target. Visual contextual processing and topographical representation of visual scenes are thus strongly and directly related to Double Decision performance.

The inferior parietal lobule (IPL), containing the temporoparietal junction is likewise associated with spatial orientation and visual target search. The IPL has been implicated in the fronto-parietal control network (FPCN), responsible for attention and control related to cue-related feedback and stimuli (Dosenbach et al., 2007). Successful Double Decision performance is dependent on the ability to attend to brief presentation of complex visual stimuli and to adapt search strategies based on feedback. As such, the nodes of the FPCN network in particular appear to be heavily implicated in the current study, as significant degradations in the inferior parietal lobule were associated with poorer Double Decision performance. Decreased cortical thickness within the IPL in the present study corroborate previous research on UFOV performance and cortical thickness declines in an ROI-based analysis (Schmidt et al., 2016).

### Right Occipital Lobe

The lateral occipital gyrus, or lateral occipital complex, is centrally linked to vision, and is particularly activated during fMRI tasks of object recognition (Grill-Spector et al., 2001). Interestingly, it is strongly activated during viewing of objects, rather than textures or faces, and appears to be preferentially activated in viewing familiar objects, rather than novel objects (Grill-Spector et al., 2001). This area was strongly implicated in the current study, with cortical thickness declines comprising 31.96% of the entire lateral occipital gyrus. Given the short duration of stimuli (on the order of milliseconds), this preferential activation in the lateral occipital gyrus for viewing familiar objects may aid in processing speed performance. Notably, this cortical area has numerous feed-forward projections spanning to higher cortical areas, such as the orbitofrontal cortex, and is associated with object recognition tasks (Grill-Spector et al., 2001; Kravitz et al., 2014). Both the lateral occipital gyrus and lateral orbitofrontal cortex are both implicated in the present study and are likely due to the object recognition aspect of the Double Decision task.

The lingual gyrus is heavily involved in vision, particularly in perception, encoding and recognition of visual targets and their orientation in space (Gayet et al., 2017). Recent fMRI research has also suggested this area is associated with complex cognitive tasks, such as tasks measuring working memory involving visual stimuli and inhibition of motor response of visual distractors (Gayet et al., 2017; Zhang et al., 2016). As such, the association between cortical thinning in this area and poorer Double Decision performance may be attributed to the visual nature of the task.

### Potential Implications of Results

#### Comparison to Other Speed of Processing Studies

Looking at a broader range of speed of processing tasks, decreased cortical volume (comprised of both cortical surface area and thickness) within the bilateral precentral gyrus, bilateral inferior frontal gyrus, left superior frontal gyrus, superior parietal regions and middle frontal gyrus was associated with poorer speed of processing performance in prior studies (Chee et al., 2009; Hong et al., 2015). The present study found several related findings including the right precentral gyrus and right superior parietal lobule but failed to replicate this finding in the left precentral gyrus, bilateral inferior frontal gyrus, left superior frontal gyrus and middle frontal gyrus. Additionally, several studies to date have suggested that cortical volume within hippocampal regions are especially influential in speed of processing performance, accounting for a significant variance in speed of processing scores (Papp et al., 2014; O’Shea et al., 2016; Tsapanou et al., 2019). The current study supports these findings. We observed significant cortical thinning in over a third of the parahippocampal gyrus in relation to Double Decision scores, suggesting the hippocampus and parahippocampal gyrus may be important in speed of processing performance. Future research should continue to assess patterns of cortical volumetric decline in a variety of speed of processing paradigms.

#### Comparison to Prior Findings in the UFOV/Double Decision Task

In a prior study, eight ROIs were implicated in a task-based UFOV fMRI task: anterior cingulate cortex, anterior insula, DLPFC, inferior parietal lobule, supplementary motor area (SMA), and thalamus. Specifically, these regions were activated when performing the UFOV task, and activation in these ROIs were reduced following a UFOV intervention (Ross et al., 2018). Notably, we found significant cortical thickness declines in a number of these regions associated with poorer Double Decision performance, including the anterior insula (AI), inferior parietal lobule, temporoparietal junction (within the IPL) and visual cortex.

Only one study to date has looked at the structural neural correlates of the UFOV task. Schmidt and colleagues found that poorer UFOV performance was associated with decreased cortical thickness in nodes associated with the frontoparietal control network (FPCN), specifically the intraparietal sulcus, frontal cortex, portions of the precentral gyrus, precuneus, midcingulate, inferior parietal lobule and dorsolateral prefrontal cortex. In the present study, we found significant cortical thickness in the right inferior parietal lobule and right precentral gyrus.

Although the Double Decision task employs additional components of visual search and distractors not found in the UFOV task used by Ross, Schmidt and colleagues, this overlap may suggest that cortical thickness declines in these ROIs may be mediating Double Decision performance. Furthermore, these cortical thickness declines may influence BOLD activation patterns in an in-scanner UFOV task. Further examining associations between structural neural correlates, BOLD fMRI, and functional connectivity networks may elucidate neural underpinning of this task and modifying cognitive interventions utilizing the Double Decision task.

### Potential Involvement of the Cingulo-Opercular Network (CON) and Fronto-Parietal Control Network (FPCN)

The present study found that cortical thickness was associated with Double Decision scores in a wide array of right hemisphere ROIs, including the fusiform gyrus, lingual gyrus, parahippocampal gyrus, insula, superior, transverse, and inferior temporal gyrus, temporal pole, precentral gyrus, inferior parietal lobule, lateral orbitofrontal gyrus and lateral occipital gyrus. Perhaps not surprisingly, the significant ROIs in the current study correspond to attention networks in the brain, specifically the cingulo-opercular network (CON) and fronto-parietal control network (FPCN) (Yeo et al., 2011).

The cingulo-opercular network (CON) is a predominately right lateralized network, comprised of the right orbitofrontal cortex, anterior insula (AI) and right temporoparietal junction (contained within the right inferior parietal lobule) (Fox et al., 2006). This network is heavily implicated in target detection and task maintenance, and regions within this network are activated when salient targets unexpectedly appear (Corbetta et al., 2000; Fox et al., 2006). Persistent CON activation is also associated with attention, error processing, and rule maintenance, all of which are implicated in the Double Decision task (Dosenbach et al., 2007). Indeed, the role of regions within the CON network in the present study is supported by work from Ross et al., who assessed functional connectivity between numerous ROIs following a UFOV cognitive training intervention (Ross et al., 2018). Of these ROIs, the orbitofrontal cortex and AI demonstrated strengthened functional connectivity and demonstrated increased BOLD activation with better UFOV performance (Ross et al., 2018). Additionally, cortical thickness declines in these regions were significantly associated with poorer Double Decision performance in the present study.

The fronto-parietal control network (FPCN) is a functional connectivity network, comprised of the inferior parietal lobule, inferior parietal sulcus, precuneus and frontal cortex (Dosenbach et al., 2007). This network is responsible for active attention processes, such as information integration and flexibility in cognitive processes (Dosenbach et al., 2007). Furthermore, the decreased cortical thickness findings within the current study are consistent with previous research investigating cortical thickness and UFOV performance in an ROI based analysis (Schmidt et al., 2016). Specifically, Schmidt and colleagues found significant cortical thickness declines in ROIs within the FPCN, such as the inferior parietal lobule, frontal cortex, and intraparietal sulci (Schmidt et al., 2016). This converging evidence may highlight the potential contribution of the FPCN in UFOV and Double Decision performance.

### Limitations and Future Directions

The present study assessed cortical thickness within older adults aged 65–88 years. However, this age range is relatively restricted, and may not generalize to a larger age range of adults. The present study also investigated associations between the Double Decision task and cortical thickness in healthy older adults. Thus, the current study is unable to assess how these cortical regions are affected in neurodegenerative disorders in relation to speed of processing tasks. Future work should expand into a broader population to assess how disparities in cortical thickness may be influenced by age or neurodegenerative disease state and Double Decision performance.

This study found significant, widespread cortical thickness declines in relation to poorer Double Decision scores, but not cortical surface area. Although cortical thickness and cortical surface area together comprise overall cortical volume, these two components have distinct cellular organizations and implications in aging (Hartberg et al., 2011; Storsve et al., 2014). Cortical surface area is thought to be a proxy of structural integrity and complexity of gray matter and declines gradually throughout the lifespan (Lemaitre et al., 2012). Cortical thickness, on the other hand, is a measure of neuronal density within a particular region of the brain, and significant cortical thickness declines occur primarily within neurodegenerative diseases states (Dickstein et al., 2007). In contrast, gradual cortical thickness declines seen within healthy aging are likely due to decreased dendritic arborization, shortened dendritic spines and shrinkage of neurons, rather than widescale neuronal death (Fjell et al., 2009; Fjell and Walhovd, 2010). However, alterations in both cortical thickness and cortical surface area have been separately shown to correlate with cognitive declines (Hartberg et al., 2011; Lemaitre et al., 2012; Nissim et al., 2017). Indeed, another studies assessing UFOV performance have demonstrated decreased cortical thickness in many of the same regions as the current study (Schmidt et al., 2016). It may be possible that the characteristics of decreased cortical thickness, such as decreased dendritic arborization or shortened dendritic spines, mediate performance on speed of processing tasks, including the Double Decision task. Additionally, the cross-sectional nature of this study does not allow us to evaluate how longitudinal alteration in cortical thickness are associated with changes in Double Decision performance throughout the aging process. As this is one of the first studies assessing structural neural correlates of UFOV or Double Decision performance, longitudinal studies are needed to further elucidate the impact of cortical thickness changes on Double Decision performance.

The present study focused on cortical alterations in thickness and surface area related to Double Decision performance. Although we were able to establish the relative percent of individual ROIs encompassed within the significant clusters, this does not necessarily correlate to its relative contributions to task performance. For instance, although the percent of decreased cortical thickness in relation to Double Decision was greater in the right insula (25.75% of total ROI) than in the right inferior temporal gyrus (4.48% of total ROI), we cannot say that cortical thickness declines in the insula contribute more to Double Decision scores than the inferior temporal gyrus. It may be that the small portion of the right inferior temporal gyrus provides more significant contributions to Double Decision scores, however, this cannot be elucidated from the current study. Nevertheless, assessment of ROI overlap within significant clusters allows us to focally determine areas within large cortical regions that are associated with Double Decision performance. Additionally, although these areas are related to Double Decision performance, we cannot assess which of these significant findings are functionally activated during Double Decision performance in the present study. Future studies should include the use of multi-modal imaging techniques, such as task-based fMRI and resting-state functional connectivity, to assess both the structural and functional neural correlates of the Double Decision task.

## Conclusion

This study provides unique insights into the structural neural correlates of the Double Decision task. Notably, we found that decreased cortical thickness in the right hemisphere is associated with poorer Double Decision performance within several areas across the cortex, including the lateral orbitofrontal gyrus, precentral gyrus, superior, transverse and inferior temporal gyrus, superior and inferior parietal lobule, the lateral occipital gyrus and the lingual gyrus. Given this task’s wide success in an extensive array of cognitive training programs, this study emphasizes unique cortical regions of the Double Decision task using a focal vertex-wise approach, potentially allowing for more targeted cognitive interventions (e.g., transcranial direct current stimulation) to improve speed of processing performance through enhanced neuroplasticity.

## Data Availability Statement

The data are managed under the data sharing agreement established with NIA and the parent R01 clinical trial Data Safety and Monitoring Board in the context of an ongoing Phase III clinical trial (ACT study, R01AG054077). All trial data will be made publicly available 2 years after completion of the parent clinical trial, per NIA and DSMB agreement. Requests for baseline data can be submitted to the ACT Publication and Presentation (P&P) Committee and will require submission of a data use, authorship, and analytic plan for review by the P&P committee (ajwoods@phhp.ufl.edu).

## Ethics Statement

The Institutional Review Board (IRB) at the University of Florida and University of Arizona reviewed and approved this study. Prior to any study procedures, all participants provided written informed consent. The study protocol was carried out in accordance with the Declaration of Helsinki, and the University of Florida and University of Arizona Institutional Review Board reviewed and approved all procedures in this study. All study participants were healthy older adults.

## Author Contributions

JK, NE, HH, EB, NN, GA, and AW contributed text to the manuscript. JK, AO’S, AA, and AW performed data analysis. EP, GA, and AW contributed to manuscript revisions. All authors provided edits and approved the final version of the manuscript.

## Conflict of Interest

The authors declare that the research was conducted in the absence of any commercial or financial relationships that could be construed as a potential conflict of interest.

## Footnotes

1 www.brainhq.com
